# Supplementary material for: Effects of the CYP3A inhibitors, voriconazole, itraconazole, and fluconazole on the pharmacokinetics of osimertinib in rats
Source: PeerJ. 2023 Aug 10;11:e15844. doi: 10.7717/peerj.15844 (PMC10423561; doi:10.7717/peerj.15844)
Supplement: Supplemental Information 1 [file peerj-11-15844-s001.pdf]

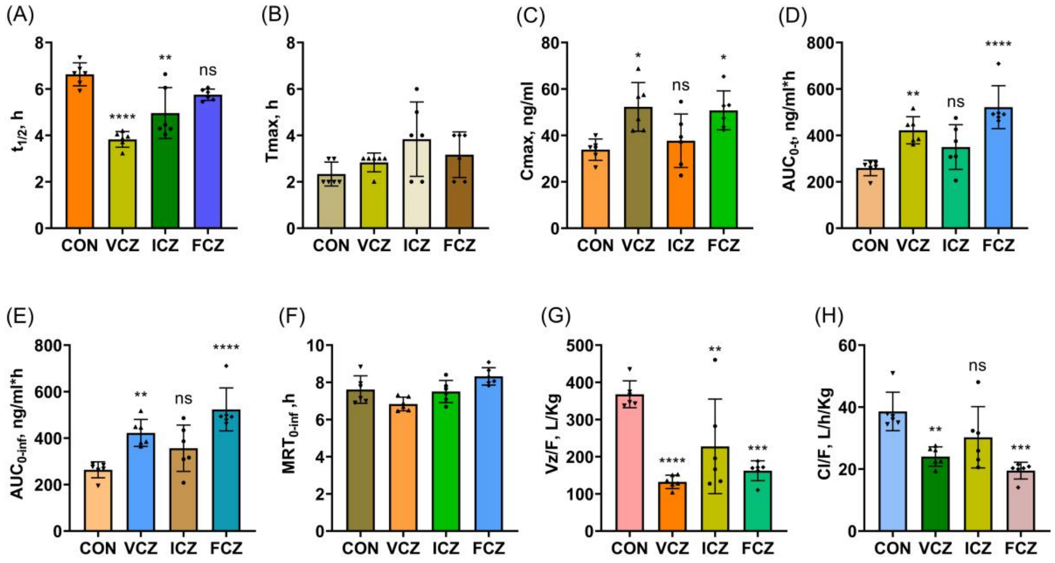

Figure S1. The results of Tukey test after oral of OSIM (10 mg/kg) for (A) $t_{1/2}$, (B) T_{max}, (C) C_{max}, (D) AUC_{0-t}, (E) AUC_{0-inf}, (F) MRT_{0-inf}, (G) Vz/F and (H) Cl/F in the control (CON), voriconazole (VCZ), itraconazole (ICZ) and fluconazole (FCZ) groups (Mean ± SD, n = 6).

Table S1. Mass spectrometry (MS) parameters of osimertinib (OSIM) and nilotinib (IS) are optimized by LC-MS/MS.

MS Parameters	Osimertinib	Nilotinib
Parent ion (m/z)	500	530
Product ion (m/z)	72.1	289.1
Declustering potential (V)	153	151
Collision energy (eV)	35	41
Ion-Spray Voltage	5500	
Source Temperature	550	
Curtain Gas	30	
Entrance Potential	10	
Dwell Time	100	
Collision Cell Exit Potential	6	
Collision Gas	9	
Gas 1	50	
Gas 2	55	

Table S2. Precision and accuracy of osimertinib (OSIM) in rat plasma (n=5).

Concentrations (ng/mL)	Intra-day			Inter-day		
	Mean ± SD	CV (%)	RE (%)	Mean ± SD	CV (%)	RE (%)
Osimertinib						
2	1.96±0.15	7.73	-2.61	1.94±0.14	7.01	-3.22
5	5.54±0.18	3.23	10.41	5.25±0.24	4.48	4.52
200	204.91±6.32	3.08	2.05	207.70±3.50	1.69	3.44
400	355.22±10.73	3.02	-11.55	364.72±10.17	2.79	-9.18

SD, Standard Deviation; CV, Coefficient of Variation; RE, Relative Error.

Table S3. Extraction recovery and matrix effect for analytes in rat plasma (n=5).

Concentrations (ng/mL)	Extraction recovery		IS normalized matrix effect	
	Mean ± SD	CV (%)	Mean ± SD	CV (%)
Osimertinib				
5	100.67±2.52	2.50	104.63±4.37	4.17
200	96.25±1.98	2.06	105.48±4.86	4.69
400	97.63±2.24	2.30	97.26±4.54	4.67
Nilotinib				
10	107.87±5.36	4.97		

Table S4. Stability results of OSIM under four different storage conditions (n=4).

Osimertinib	LQC (5 ng/mL)		MQC (200 ng/mL)		HQC (400 ng/mL)		Relative stability (%)
	Mean ± SD	CV (%)	Mean ± SD	CV (%)	Mean ± SD	CV (%)	
Room temperature for 6h	5.40±0.16	3.03	211.16±10.78	5.11	387.49±18.71	4.83	96.49-107.48
Autosampler for 24 h	5.18±0.22	4.32	194.56±10.29	5.29	348.13±12.54	3.32	94.15-103.09
Three freeze-thaw cycles	5.32±0.34	6.42	195.09±5.67	2.91	363.62±7.40	2.03	90.54-106.03
Long-term at -30 °C for 30 days	4.47±0.12	2.63	199.05±6.97	3.5	397.22±11.70	2.95	88.96-98.91

Table S5. Evaluation of the carry-over for OSIM and IS in rat plasma (n=6).

Analytes	Area of ULOQ	Area of blank	Area of LLOQ	Interference (%)
Osimertinib	4219990.3	881	19831.5	4.44
Nilotinib	95503.8	103.8	/	0.11
